# The complete sequences and gene organisation of the mitochondrial genomes of the heterodont bivalves *Acanthocardia tuberculata *and *Hiatella arctica *– and the first record for a putative Atp*ase subunit 8 *gene in marine bivalves

**DOI:** 10.1186/1742-9994-3-13

**Published:** 2006-09-01

**Authors:** Hermann Dreyer, Gerhard Steiner

**Affiliations:** 1 Emerging Focus Molecular Biology, Department of Evolutionary Biology, University of Vienna, 1090 Vienna, Austria

## Abstract

**Background:**

Mitochondrial (mt) gene arrangement is highly variable among molluscs and especially among bivalves. Of the 30 complete molluscan mt-genomes published to date, only one is of a heterodont bivalve, although this is the most diverse taxon in terms of species numbers. We determined the complete sequence of the mitochondrial genomes of *Acanthocardia tuberculata and Hiatella arctica*, (Mollusca, Bivalvia, Heterodonta) and describe their gene contents and genome organisations to assess the variability of these features among the Bivalvia and their value for phylogenetic inference.

**Results:**

The size of the mt-genome in *Acanthocardia tuberculata *is 16.104 basepairs (bp), and in *Hiatella arctica *18.244 bp. The *Acanthocardia *mt-genome contains 12 of the typical protein coding genes, lacking the *Atpase subunit 8 *(*atp8*) gene, as all published marine bivalves. In contrast, a complete *atp8 *gene is present in *Hiatella arctica*. In addition, we found a putative truncated *atp8 *gene when re-annotating the mt-genome of *Venerupis philippinarum*. Both mt-genomes reported here encode all genes on the same strand and have an additional *trnM*. In *Acanthocardia *several large non-coding regions are present. One of these contains 3.5 nearly identical copies of a 167 bp motive. In *Hiatella*, the 3' end of the *NADH dehydrogenase subunit *(*nad*)*6 *gene is duplicated together with the adjacent non-coding region. The gene arrangement of *Hiatella *is markedly different from all other known molluscan mt-genomes, that of *Acanthocardia *shows few identities with the *Venerupis philippinarum*. Phylogenetic analyses on amino acid and nucleotide levels robustly support the Heterodonta and the sister group relationship of *Acanthocardia *and *Venerupis*. Monophyletic Bivalvia are resolved only by a Bayesian inference of the nucleotide data set. In all other analyses the two unionid species, being to only ones with genes located on both strands, do not group with the remaining bivalves.

**Conclusion:**

The two mt-genomes reported here add to and underline the high variability of gene order and presence of duplications in bivalve and molluscan taxa. Some genomic traits like the loss of the *atp8 *gene or the encoding of all genes on the same strand are homoplastic among the Bivalvia. These characters, gene order, and the nucleotide sequence data show considerable potential of resolving phylogenetic patterns at lower taxonomic levels.

## Background

Metazoan mitochondrial genomes are typically conserved in gene content and length. They are usually circular, 14 to 20 kb long, and encode for 13 proteins of the respiratory chain [cytochrome *c oxidase subunits I-III *(*cox I – cox III*), apocytochrome *b *(*cytb*), *atpase subunits 6 and 8 *(*atp6, atp8*), and *NADH dehydrogenase subunits 1–6 *and *4L *(*nad 1–6*, *nad 4L*)] and 24 RNA genes of the translation system [small (S) and large (L) subunit ribosomal RNA (rrn) and 22 transfer RNAs] [1]. The high number of possible arrangements makes it very unlikely that identical gene orders arise by chance [2]. Such a complex character combined with a low frequency of gene rearrangements is highly valuable for reconstructing palaeozoic or even pre-Cambrian phylogenetic events. Examples for this situation are Vertebrata (over 540 species sequenced) and Arthropoda (over 100 species sequenced): both show few rearrangements within the phylum [3].

In contrast, only 30 complete mitochondrial genomes of Mollusca are published: ten Gastropoda, nine Bivalvia, one Polyplacophora, two Scaphopoda and eight Cephalopoda. However, even this small taxonomic sample reveals much greater variability of gene arrangements compared to vertebrates and arthropods and notable differences in rearrangement frequencies between phyla and also within the Mollusca [3]. Whereas the order of the protein coding and the rRNA (rrn) genes in the mt genomes of the polyplacophoran *Katharina tunicata*, the vetigastropod *Haliotis rubra *and the cephalopods *Octopus vulgaris and Octopus ocellatus *are identical and the apogastropod *Ilyanassa obsoleta *and the other cephalopods can be related to them, the euthyneuran gastropods, the scaphopods and the bivalves are highly rearranged.

An additional complication in the Bivalvia, termed doubly uniparental inheritance (DUI), is the existence of distinct male and female mitochondrial lineages [4-10]. It is not clear whether this mode of inheritance is characteristic for all bivalves, or if it contributes to the accelerated rearrangement rate in this group. There are yet more special features of molluscan mt genes. Hoffmann et al. [11] described an additional *trn*-Met in *Mytilus edulis; Katharina tunicata *has two additional tRNAs [12]. Some pulmonate gastropods have unusual tRNA *s *lacking the T-stem or the D-stem, similar to nematode mt tRNAs [13]. The *atp6 *and *atp8 *genes are separated in scaphopods [14,15] and most gastropods (only the prosobranch *Littorina saxatilis*, the vetigastropd *Haliotis rubra *and the apogastropod *Ilyanassa obsoleta *have adjacent *atp6 *and *atp8*). The published heterodont and pteriomorph bivalve sequences lack the *atp8 *gene altogether. This is unusual because the *atp6 *– *atp8 *cluster is common to most animal mitochondrial genomes, often with overlapping reading frames [3]. It is, thus, not clear for which molluscan taxa and on which systematic levels mitochondrial gene order data and genomic characters like those mentioned above are phylogenetically informative.

The phylogenetic relationships of the major taxa of the heterodont bivalves are only partly resolved. Molecular phylogenetic analyses [16,17] agreed on the exclusion of the Hiatellidae from the Myoida placing this taxon close to the base of the higher Heterodonta ("unnamed clade I" in [16] fig. 3.6). The latter clade also contains the Cardiidae and Veneridae. With the complete mitochondrial sequences of one species of each Hiatellidae, Carditidae and Veneridae available for the present study we are able to test the monophyly and sistergroup relationships of the higher heterodonts, *Acanthocardia *and *Venerupis*.

## Results

### Genome size, genes, base composition and codon usage

**Figure 1:**
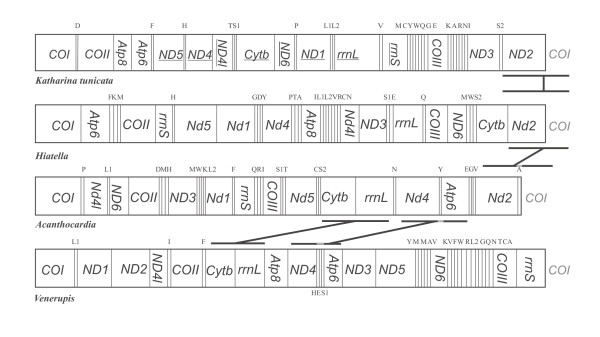
**Gene order of *Katharina*, *Acanthocardia*, *Hiatella* and *Venerupis***. Linearized representation of the mitochondrial gene arrangement in *Acanthocardia tuberculata *and *Hiatella arctica*, in comparison with the near-plesiomorphic condition in the polyplacophoran *Katharina tunicata *and the third heterodont bivalve,*Venerupis philipinarium*. Genes encoded on the opposite strand are underlined. The bars indicate identical gene junctions.

**Figure 2:**
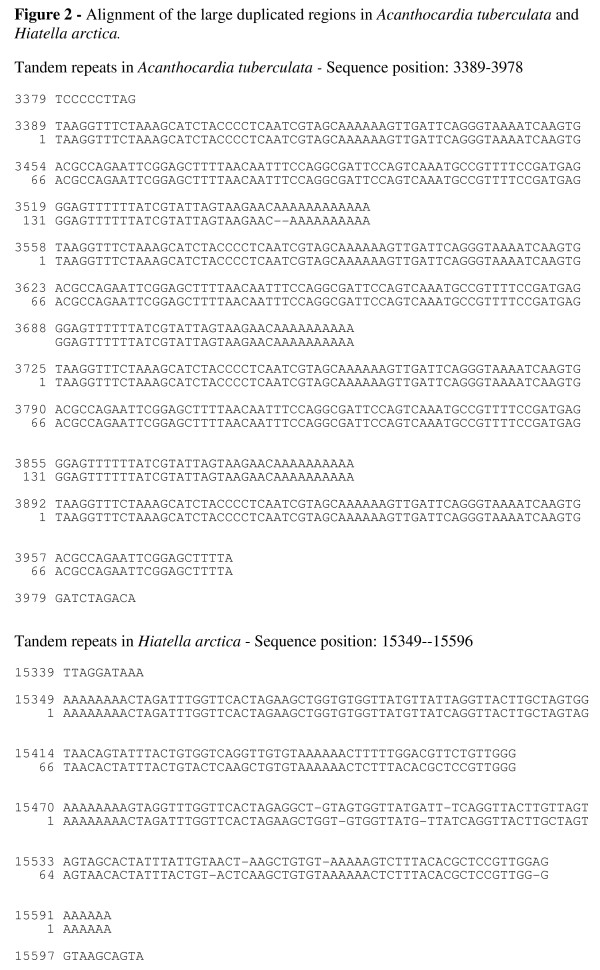
Alignment of the large duplicated regions in *Acanthocardia tuberculata *and *Hiatella arctica*.

The size of the complete mt-genome of *Acanthocardia tuberculata *is 16.104 basepairs (bp) and has an overall A+T content of 59.6 %. All genes are on one strand (Fig. 1). The *Acanthocardia *genome features 1.751 non-coding bp. The largest non-coding region (Table 1), of 1.103 bp is located between *trn-Met *and *trn-His*. It contains a 599 bp fragment composed of 3.5 nearly identical copies of a 167 bp motive (Fig. 2). This repeat has an A+T content of 60 %. The other 23 non-coding regions range between 1 and 128 bp.

**Table 1:** Non-coding regions and overlaps in the mitochondrial genome of *Acanthocardia tuberculata*

**Position**	**between**	**between**
*Non-coding*		
1594–1598	*COI/tRNA-Pro*	5
1663–1699	*tRNA-Pro/ND4l*	37
1982–2000	*ND4l/tRNA-Leu*	19
2528–2531	*ND6/COII*	4
3318–3326	*tRNA-Asp/tRNA-MetI*	9
3395–4497	*tRNA-MetI/tRNA-His*	1103
4559–4571	*tRNA-HisI/ND3*	13
4920–4927	*ND3/tRNA-MetII*	8
4992–4999	*tRNA-MetII/tRNA-Trp*	8
5064	*tRNA-Trp/tRNA-Lys*	1
5197–5226	*tRNA-Leu/ND1*	30
6139–6148	*ND1/tRNA-Phe*	10
7041–7070	*12S rRNA/tRNA-Gln*	30
8099–8104	*COIII/tRNA-SerI*	6
8174	*tRNA-SerI/tRNA-Thr*	1
8238–8293	*tRNA-Thr/ND5*	56
10005–10006	*tRNA-Cys/tRNA-SerII*	2
12521–12648	*tRNA-Asn/ND4*	128
13990–13994	*ND4/tRNA-Tyr*	5
14060–14064	*tRNA-Tyr/Atp6*	5
14777–14884	*Atp6/tRNA-Glu*	108
14944–14995	*tRNA-Glu/tRNA-Gly*	52
15067–15099	*tRNA-Val/ND2*	33
16027–16104	*ND2/COI*	78
*Overlapping*		
7132–7133	*tRNA-Gln/tRNA-Arg*	2

**Figure 3:**
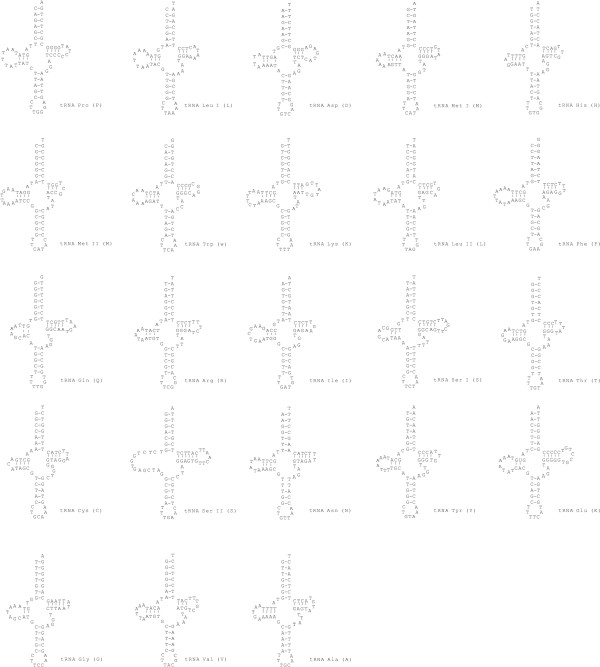
Cloverleaf structures of the 23 tRNA genes in the mitochondrial genome of *Acanthocardia tuberculata*.

All but one (*atp8*) of the 37 typical mitochondrial genes are present, with an additional copy of the *trn-Met *(Fig. 3). The *Acanthocardia *mt-genome encodes for a total of 3.647 amino acids. The most frequent codon is TTT (Phe; n = 264), followed by TTA (Leu; n = 172). An A or T nucleotide is present at the third position in 2.269 codons (61.13%). Five of the 12 protein coding genes start with ATG or ATA (Table 2), six starts with the alternative start codon ATT (Isoleucine). The *atp6 *gene starts with a GTG codon. Eight genes are terminated by TAA and four by TAG. An incomplete stop codon is inferred from the alignment of the *atp6 *gene. The genes for *trn-Gln *and *trn*-*Arg *overlap by two 2 bp.

**Table 2:** Organisation of the mitochondrial genome of *Acanthocardia tuberculata*

**Gene**	**Position**	**Strand**	**Start**	**Stop**
*COI*	*1–1593*	+	ATA	TAG
*tRNA-Pro*	*1599–1662*	+		
*ND4l*	*1700–1981*	+	ATT	TAA
*tRNA-LeuI*	*2001–2065*	+		
*ND6*	*2066–2527*	+	ATT	TAA
*COII*	*2532–3254*	+	ATT	TAA
*tRNA-Asp*	*3255–3317*	+		
*tRNA-MetI*	*3327–3394*	+		
*tRNA-His*	*4498–4558*	+		
*ND3*	*4572–4919*	+	ATG	TAA
*tRNA-MetII*	*4928–4991*	+		
*tRNA-Trp*	*5000–5063*	+		
*tRNA-Lys*	*5065–5132*	+		
*tRNA-LeuII*	*5133–5196*	+		
*ND1*	*5227–6138*	+	ATG	TAG
*tRNA-Phe*	*6149–6216*	+		
*12S rRNA*	*6217–7040*	+		
*tRNA-Gln*	*7071–7133*	+		
*tRNA-Arg*	*7132–7196*	+		
*tRNA-Ile*	*7197–7261*	+		
*COIII*	*7262–8098*	+	ATG	TAA
*tRNA-SerI*	*8105–8173*	+		
*tRNA-Thr*	*8175–8237*	+		
*ND5*	*8294–9940*	+	ATT	TAA
*tRNA-Cys*	*9941–10004*	+		
*tRNA-SerII*	*10007–10074*	+		
*Cytb*	*10075–11232*	+	ATT	TAG
*16S rRNA*	*11233–12455*	+		
*tRNA-Asn*	*12456–12520*	+		
*ND4*	*12649–13989*	+	ATA	TAA
*tRNA-Tyr*	*13995–14059*	+		
*Atp6*	*14065–14776*	+	GTG	T incomplete
*tRNA-Glu*	*14885–14943*	+		
*tRNA-Gly*	*14996–15007*	+		
*tRNA-Val*	*15008–15066*	+		
*ND2*	*15100–16026*	+	ATT	TAA
*tRNA-Ala*	*16029–16092*	+		

**Figure 4:**
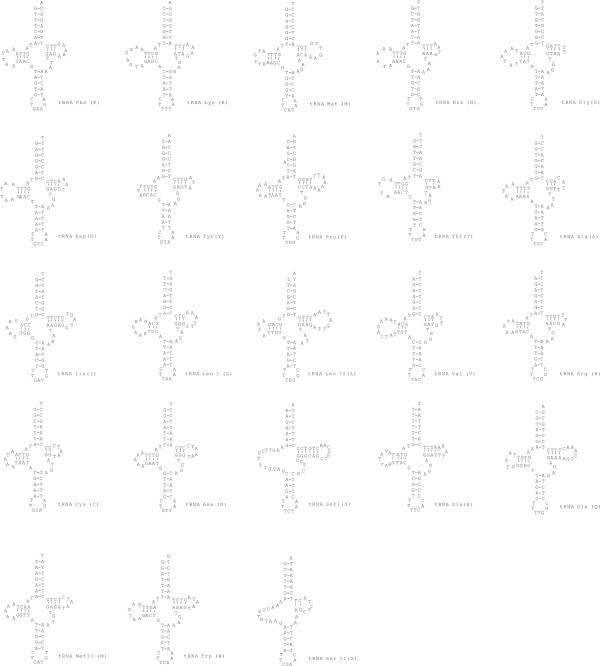
Cloverleaf structures of the 23 tRNA genes in the mitochondrial genome of *Hiatella arctica*.

The mt-genome of *Hiatella arctica *is 18.204 bp in length and has an A+T content of 66.35 %. As in *Acanthocardia*, all genes are on the same strand (Fig. 1). The longest of the 30 non-coding regions (Tab 3) has 614 bp and is located between the genes for *trn-Ala *and *atp8*. The others range between 1 and 376 bp in length.*Hiatella *has two copies of a 121 bp motive (Fig. 4) starting in the 3' end of the *nad6 *gene and extending into the non-coding region before the *tRNA-Trp *gene. The genes for *trn-Leu I *and *trn*-*LeuII *overlap by one nucleotide.

**Table 3:** Non-coding regions and overlaps in the mitochondrial genome of *Hiatella arctica*

**Position**	**between**	**Length**
*Non-coding*		
1654–1819	*COI/Atp6*	166
2603–2609	*Atp6/tRNA-Phe*	7
2674–2678	*tRNA-Phe/tRNA-Lys*	5
2743	*tRNA-Lys/tRNA-Met*	1
2809	*tRNA-Met/COII*	1
3894–4270	*COII/12S rRNA*	376
5235–5294	*tRNA-His/ND5*	60
6963–6983	*ND5/ND1*	21
7929	*ND1/tRNA-Gly*	1
7992–7998	*tRNA-Gly/tRNA-Asp*	7
8063–8105	*tRNA-Asp/tRNA-Tyr*	43
8166	*tRNA-Tyr/ND4*	1
9529–9533	*ND4-tRNA-Pro*	5
9601–9621	*tRNA-Pro/tRNA-Thr*	21
9682–8690	*tRNA-Thr/tRNA-Ala*	9
9753–10366	*tRNA-Ala/Atp8*	614
10527–10730	*Atp8/tRNA-Ile*	204
10796–10800	*tRNA-Ile/tRNA-LeuI*	5
10932–10933	*tRNA-Leu/tRNA-Val*	2
11062	*tRNA-Arg/tRNA-Cys*	1
11124–11129	*tRNA-Cys/tRNA-Asn*	6
11094–11311	*tRNA-Asn/Nd4l*	218
11612–11672	*Nd4l/Nd3*	61
12030–12055	*ND3/tRNA-SerI*	26
13707–13924	*tRNA-Gln/COIII*	218
14753–14878	*COIII/ND6*	126
15482–15722	*ND6/tRNAMetII*	241
15855–15875	*tRNA-Trp/tRNA-SerII*	21
15931–16000	*tRNA-SerII/Cytb*	70
18197–18244	*ND2/COI*	48
*Overlapping*		
10864	*tRNA-LeuI/tRNA-LeuII*	1

The *Hiatella *mt-genome contains all 37 mitochondrial genes including *atp8 *and a second copy of the *trn-Met *(Fig. 4). A total of 3.985 amino acids are encoded. As in *Acanthocardia*, the most frequent codons are TTT (Phe; n = 359) and TTA (Leu; n = 284). A or T are present in 2.873 third codon positions (72.09 %). Seven of the 13 protein coding genes start with ATA, the other six genes with ATG. The codon ATT terminates seven, and the codon ATG four protein coding genes (Tab 4). Truncated stop codons (T) are inferred for the *atp8 *and the *coxII *genes.

**Table 4:** Organisation of the mitochondrial genome of *Hiatella arctica*

**Gene**	**Position**	**Strand**	**Start**	**Stop**
*COI*	1–1653	+	ATG	TAG
*Atp6*	1820–2602	+	ATG	TAA
*tRNA-Phe*	2610–2673	+		
*tRNA-Lys*	2679–2743	+		
*tRNA-Met*	2745–2808	+		
*COII*	2810–3893	+	ATA	T incomplete
*12S rRNA*	4271–5171	+		
*tRNA-His*	5172–5234	+		
*ND5*	5295–6962	+	ATA	TAA
*ND1*	6984–7928	+	ATA	TAA
*tRNA-Gly*	7930–7991	+		
*tRNA-Asp*	7999–8062	+		
*tRNA-Tyr*	8106–8165	+		
*ND4*	8167–9528	+	ATA	TAA
*tRNA-Pro*	9534–9600	+		
*tRNA-Thr*	9622–9681	+		
*tRNA-Ala*	9691–9752	+		
*Atp8*	10367–10526	+	ATG	T incomplete
*tRNA-Ile*	10731–10795	+		
*tRNA-LeuI*	10801–10864	+		
*tRNA-LeuII*	10864–10931	+		
*tRNA-Val*	10934–10998	+		
*tRNA-Arg*	10999–11061	+		
*tRNA-Cys*	11063–11123	+		
*tRNA-Asn*	11130–11193	+		
*ND4l*	11312–11611	+	ATG	TAG
*ND3*	11673–12029	+	ATA	TAG
*tRNA-SerI*	12056–12125	+		
*tRNA-Glu*	12126–12191	+		
*16S rRNA*	12192–13638	+		
*tRNA-Gln*	13639–13706	+		
*COIII*	13925–14752	+	ATA	TAA
*ND6*	14879–15481	+	ATG	TAG
*tRNA-MetII*	15723–15788	+		
*tRNA-Trp*	15789–15854	+		
*tRNA-SerII*	15876–15930	+		
*Cytb*	16001–17155	+	ATA	TAA
*ND2*	17156–18196	+	ATG	TAA

### Phylogenetic analysis of nucleotide and protein coding sequences

**Figure 5:**
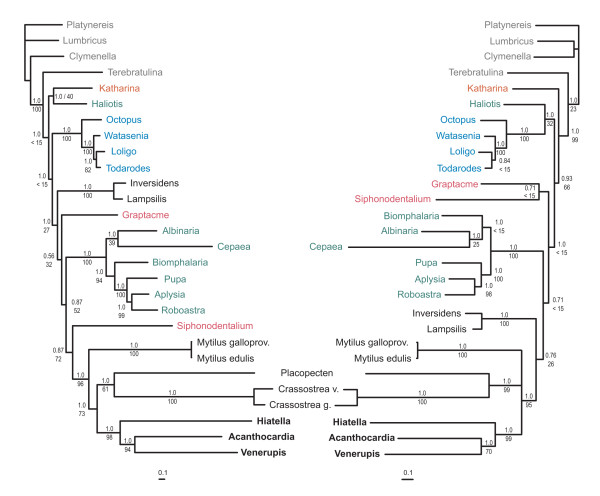
**Phylogenetic analyses**. Bayesian trees of the amino acid sequences of all protein coding genes (left) and nucleotide sequences of the protein coding genes and the *rrnL *gene (right). Bivalve species are in black font (Heterodonta in bold), Gastropoda in green, Cephalopoda in blue, Scaphopoda in magenta, Polyplacophora in red and the outgroup taxa in grey font. Posterior probabilities (above) and parsimony bootstrap values (below) are given for each branch.

The concatenated amino acid alignment of 28 species (Tab 5) consists of 5.004 positions of which 3.085 are parsimony-informative. The corresponding nucleotide alignment including the rrnL sequences has 16.862 positions in total, 11.854 without 3^rd ^codon positions, of which 7.130 are parsimony-informative. The Bayesian analyses resulted in almost fully resolved trees (Fig. 5) with total marginal -lnL of 156.458,59 for the amino acid data and 209.800,11 for the nucleotide data (arithmetic means). Most branches have posterior probabilities of 1.0. The deeper nodes tend to be less supported. The parsimony analyses of both data sets yielded a single most parsimonious tree each (amino acid data: tree length 31.163, consistency index 0.5819, rescaled consistency index 0.2719; nucleotide data: tree length 49.859, consistency index 0.3440, rescaled consistency index 0.1366; trees not shown). All Bayesian and parsimony analyses recover the three heterodont species as a robust monophylum. *Acanthocardia *is the sister taxon of *Venerupis *with high to moderate support (fig 5). The Pteriomorpha are resolved as monophyletic from the nucleotide data in the parsimony analysis only. In most analyses the two unionid species are separated from the remaining bivalves and placed in a more basal position in the tree. Only the Bayesian tree of the nucleotide data resolves monophyletic Bivalvia, although with low support (posterior probability 0.76). This is also the only tree showing monophyletic Scaphopoda and Pulmonata. The unstable position of the vetigastropod *Haliotis*, near the base of the molluscan clade renders the Gastropoda diphyletic in all analyses. Cephalopoda are always robustly supported, and only the parsimony analysis of the amino acid data fails to resolve Mollusca as monophyletic. The exclusion of highly variable alignment positions using GBLOCKS had no effect on the topologies of the trees and brought only minimal changes in branch support.

**Table 5:** List of taxa used in the phylogenetic analysis

***Taxon***	***Classification***	***GenBank Accession number***
Outgroup		
*Terebratulina retusa*	Brachiopoda	AJ245743
*Lumbricus terrestris*	Annelida, Clitellata	U24570
*Platynereis dumerilii*	Annelida, Polychaeta	AF178678
*Clymenella torquata*	Annelida, Polychaeta	AY741661
Polyplacphora		
*Katharina tunicata*	Mollusca, Polyplacophora, Neocoleida	U09810
Gastropoda		
*Haliotis rubra*	Mollusca; Gastropoda, Orthogastropoda, Vetigastropoda	AY588938
*Aplysia californica*	Mollusca; Gastropoda, Orthogastropoda, Apogastropoda	AY569552
*Pupa strigosa*	Mollusca; Gastropoda, Orthogastropoda, Apogastropoda	AB028237
*Roboastra europaea*	Mollusca; Gastropoda, Orthogastropoda, Apogastropoda	AY083457
*Biomphalaria glabrata*	Mollusca; Gastropoda, Pulmonata, Basammatophora	AY380531
*Albinaria caerulea*	Mollusca; Gastropoda, Pulmonata, Stylommatophora	X83390
*Cepaea nemoralis*	Mollusca; Gastropoda, Pulmonata, Stylommatophora	U223045
Scaphopoda		
*Graptacme eborea*	Mollusca, Scaphopoda, Dentaliida	AY484748
*Siphonodentalium lobatum*	Mollusca, Scaphopoda, Gadilida	AY342055
Cephalopoda		
*Octopus vulgaris*	Mollusca, Cephalopoda, Coleoidea, Neocoleoidea, Octopodiformes	AB158363
*Loligo bleekeri*	Mollusca, Cephalopoda, Coleoidea, Neocoleoidea, Decapodiformes	AB029616
*Todarodes pacificus*	Mollusca, Cephalopoda, Coleoidea, Neocoleoidea, Decapodiformes	AB158364
*Watasenia scintillians*	Mollusca, Cephalopoda, Coleoidea, Neocoleoidea, Decapodiformes	AB086202
Bivalvia		
*Placopecten magellanicus*	Mollusca, Bivalvia, Pteriomorphia, Pectinoida	DQ088274
*Mytilus galloprovincialis*	Mollusca, Bivalvia, Pteriomorphia, Mytiloida	AY497292
*Mytilus edulis*	Mollusca, Bivalvia, Pteriomorphia, Mytiloida	AY484747
*Crassostrea gigas*	Mollusca, Bivalvia, Pteriomorphia, Ostreoida	AF177226
*Crassostrea virginica*	Mollusca, Bivalvia, Pteriomorphia, Ostreoida	AY905542
*Lampsilis ornata*	Mollusca, Bivalvia, Palaeoheterodonta, Unionida	AY365191
*Inversidens japanensis*	Mollusca, Bivalvia, Palaeoheterodonta, Unionida	AB055625
*Venerupis phillipinarium*	Mollusca, Bivalvia, Heterochonchia, Veneroida	AB065375
*Acanthocardia tuberculata*	Mollusca, Bivalvia, Heterochonchia, Veneroida	DQ_632743 this study
*Hiatella arctica*	Mollusca, Bivalvia, Heterochonchia, Myoida	DQ_632742 this study

## Discussion

Base frequencies, codon usage and amino acid frequencies in the mt genome of *Acanthocardia tuberculata *compare well with that of other bivalves. For instance, the A+T content of 59.6 % is similar to that *Inversidens *(57.2), *Lampsilis *(62.3), *Crassostrea gigas *(63.4%) and *Crassostrea virginica *(62.8%), *Placopecten *(55.7) and *Mytilus *(61.8%), but it is lower than that of *Venerupis *(69.7%) and *Hiatella arctica *(66.4).

The functional and selective significance of the duplicated regions in *Acanthocardia *is unclear. Tandem repeats are also present in other bivalve mitochondrial genomes: *Venerupis*, e.g., has four tandem repeats of 203 bp between the *nad2 *and the *nad4l *genes [9]; *Placopecten *has seven repeats of a 79 bp motive between *trn- Asn *and *trn-Glu *and two repeats of 1.435 bp between *nad6 *and *trn-Met *[18]. More unusual is the duplication in *Hiatella *starting 12 bases upstream of the 3' end of the *nad6 *gene. Although the copies are 79 % identical the second repeat has no open reading frame. It is likely that this non-functional copy of the coding part accumulated substitutions more rapidly due to relaxed selection and, thus, lost the reading frame. Nearly identical duplications of complete genes occur in mt-genomes of the cephalopods *Watasenia *and *Todarodes *[19,20].

*Acanthocardia *and *Hiatella *mt genomes encode 23 transfer RNA genes which can be folded in a typical secondary structure. Both genomes have an additional tRNA for Methionine. A second Methionine tRNAs is present in the bivalves *Mytilus edulis *[11], *Mytilus galloprovincialis *[7], *Crassostrea virginica *[21], *Placopecten magellanicus *[18] and *Venerupis phillipianarum *[9]. Overlaps of tRNA genes as observed in *Acanthocardia *and *Hiatella *are a common feature in mt-genomes [1].

The *atp6 *gene of *Acanthocardia *lacks a Methione or Isoleucine at the putative 5' end and a complete stop codon. The first ATN codon is 48 bp downstream of the putative point of start as inferred from the alignment of the molluscan *atp6 *genes. The assumed start codon is GTG as in the *nad 2 *gene of polyplacophore *Katharina tunicata *[12]. Truncated stop codons like in the *Acanthocardia atp6 *and the *Hiatella co II *and the *atp8 *genes require the inference of the ends of the genes from the alignment with other species. The completion of truncated stop codons by polyadenylation after transcript processing was described by Ojala [22].

**Figure 6:**
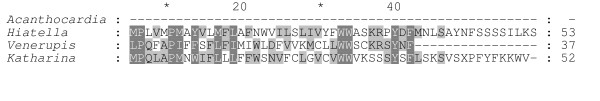
Alignment of the *atp8 *genes of *Hiatella arctica, Venerupis philippinarum *and *Katharina tunicata*.

*Hiatella arctica *is the first marine bivalve reported to have a complete *atp8 *gene consisting of 53 amino acids. The alignment of this *atp8 *gene (Fig. 6) shows a *Methionine *at the start and a truncated stop codon T. We also identified a putative *atp8 *gene in the mt-genome of *Venerupis*, between the genes *rrnL *and *nad4 *at positions 5.974 to 6.088. Although this region was annotated as part of the *rrnL *by the authors [9], it represents an open reading frame encoding for only 37 amino acid positions. It starts with *Leucine *instead of *Methionine*, but ends with a complete stop codon. The more conserved 5' region of the gene resembles other molluscan atp8 genes in amino acid sequence (Fig. 6) and in the hydrophilicity profile. The positively charged 3' region of the gene, which is known to vary greatly in length and composition [23,24], is reduced to a few residues in *Venerupis*. This is confirmed by the alignment of the amino acid sequence corresponding to the conserved *atp8 *profiles in other metazoans [25]. It remains open, however, whether this gene is functional. Dreyer and Steiner [15] reported a comparably short *atp8 *gene for the scaphopod *Siphonodentalium lobatum*. Serb and Lydeard [26] discuss a non functional version of the *atp8 *gene in the freshwater mussel *Inversidens*, and Milbary and Gaffney [21] describe a potential remnant of the *atp8 *gene in the eastern oyster *Crassostrea virginica*.

Many metazoan mt genomes have neighbouring *atp6 *and *atp8 *genes on the same strand. This arrangement is likely to be selected for, if the uncleaved transcripts are co-translated [25,2]. Several taxa lacking this gene arrangement in the mt genome, e.g. Plathyhelminthes, Nematoda, Annelida, Sipunculida, the brachipods *Laqueus *[27] and *Terebratalia *[28], and, among Mollusca, Bivalvia and Scaphopoda. Of these genomes, Plathyhelminthes, Nematoda except for *Trichinella*, and the pteriomorph bivalves lack *atp8 *altogether. The disparate distribution of this feature clearly indicates that the loss of the *atp6 *– *atp8 *coupling and the loss of *atp8 *occurred several times independently in metazoan evolution. This is corroborated by finding truncated *atp8 *genes separated from the *atp6 *gene in the nematode *Trichinella *and in the scaphopod *Siphonodentalium*. It is possible that this situation represents an evolutionary stepping stone from the fully functional *atp6 *– *atp8 *coupling, via decoupled but complete genes like in annelids and the scaphopod *Graptacme*, and the complete loss of *atp8*.

The location of all mt-genes on the same strand, as in *Acanthocardia *and *Hiatella*, is uncommon among Metazoa, but is reported for several taxa [28] including all published marine bivalves. Only in the unioid freshwater bivalves *Lampsilis ornata *and *Inversidens japanensis *genes are located on both strands. Under the Heteroconchia concept postulating a sister group relationship of Unionida and Heterodonta, the "all-on-one-strand" situation either evolved independently in Heterodonta and Pteriomorph or was lost in the Unionida.

Comparing the gene arrangements of *Acanthocardia *and *Venerupis *no identities are apparent, if the tRNA genes are included. The tRNAs are more variable because the secondary structure allows them to translocate more frequently [12]. Even after excluding the tRNAs from the comparison the two mt-genomes show few identical gene junctions. These are limited to the block containing the *Cytb *– *rrnL *– *nad4 *– *atp6 *genes in *Acanthocardia*, although this is interrupted by the putative *atp8 *gene in *Venerupis*. This gene order may be inherited from the common ancestor of *Acanthocardia *and *Venerupis*, with the apomorphic loss of *atp8 *in *Acanthocardia*. The mt-genome of *Hiatella *appears almost completely rearranged. Only the neighbourhood of the *nad2 *and *cox I *genes is present in other molluscs like *Katharina*, *Haliotis *and *Octopus *and may represent a plesiomorphic trait.

The mitochondrial genome sequence data confirm previous results [16,17] on the monophyly of Cardiidae and Veneridae relative to the Hiatellidae. Their common branch and the heterodont clade are robustly supported in all analyses. Similarly, the clade uniting Heterodonta and Pteriomorpha is well supported, although to the exclusion of the unionid branch. This is in accordance with the topology of Giribet and Distel [16] but contrasts that of Waller [29] and Steiner and Hammer [30] supporting the Heteroconchia clade (Unionida + Heterodonta). The Bivalvia clade is resolved by the Bayesian analysis of the nucleotide data only. This may indicate the higher potential of recovering correct topologies by this method or the superiority of nucleotide substitution models over amino acid substitution models or a combination of these factors. Note that the Bayesian nucleotide analysis also succeeds in resolving the pteriomorph, scaphopod, and pulmonate branches.

What could cause the unexpected position of the Unionida rendering the Heteroconchia diphyletic? In both amino acid and nucleotide-based trees the unionid species have conspicuously shorter branches compared to the other bivalves. Although the present data set is not large enough for statistical assessment, such obvious differences in substitution rates may cause phylogenetic analyses to find incorrect trees, as previously documented for Bivalvia [30]. In addition to lower substitution rates, different substitution patterns in the unionids may confound phylogenetic analyses. All bivalve mt-genomes have the genes encoded on the same strand, except for those of the unionids where three to four genes are encoded on the opposite strand. Due to the asymmetric replication process the strands show different substitution skews. Hassanin *et al*.[31] showed that skew differences may influence phylogenetic analyses.

The mitochondrial gene order in the Bivalvia is too divergent and the present taxon set too small to make use of this character set for phylogenetic inference at this point. In addition, the substitution models for phylogenetic inference presently do not take strand specific patterns into account. Similarly, gene rearrangement models are limited to one type of rearrangement only, either translocation or inversion. However, with a growing set of mt-genomes – their nucleotide and gene sequences – we are likely to enhance our understanding of patterns and modes of nucleotide substitutions and gene rearrangements. This will help to improve phylogenetic reconstructions by refining the models for these evolutionary processes. Improved taxon sampling and refined phylogenetic inference models are likely to resolve more open questions of bivalve phylogeny and evolution than with previously used markers.

## Methods

### Material, DNA isolation, PCR, sequencing

*Hiatella arctica *(Linné, 1767) and *Acanthocardia tuberculata *(Linné, 1758) were collected in the Adriatic Sea (Rovinj, Croatia) and frozen in liquid nitrogen. Total DNA was isolated with the DNeasy Tissue Kit (Qiagen).

Partial *cox I*, *rrnL *and *rrnS *genes were amplified (*Acanthocardia rrnL *and *cox I*, *Hiatella rrnS *and *rrnL*) using the primers HCO2198/LCO 1490, 16Sfmwg/16Srmwg and 12Sai/12Sbi (Table 6). The PCR was done on a Primus 96 advanced Gradient (Peqlab) in a 30 μl reaction containing 1,5 mM Mg Cl_2_, each dNTP at 250 μM, each primer at 0,5 μM, 0,6 units Taq polymerase (Biotaq Red, Bioline) and the supplied buffer at 1× concentration. The PCR cycle conditions were: Initial denaturation step of 2 min at 94°C, 35 cycles of 30 sec denaturation at 94°C, 40 sec annealing at 48°C and 2 min (HCO2198/16Sbr) or 45 sec primer extension at 72°C followed by a final primer extension step at 72°C for 7 min. PCR products were purified with the E.Z.N.A Cycle-Pure Kit (Peqlab, Germany). PCR products were sequenced automatically with the amplification primers on an ABI-capillary-sequencer at Eurofins-Medigenomix GmbH (Martinsried, Germany). The sequences were used to design sets of long range primers (*12SrRNA, CO1, 16SrRNA *Primer see table 3) to amplify the whole mitochondrial Genome in three fragments with the TaKaRa LA Taq (Takara) on a Primus 96 advanced Gradient (Peqlab). The 50 μl reactions contained 5 μl of the supplied buffer 3 (including 22.5 mM MgCl_2_), 25 pmol of each primer, 500 μM dNTPs, 5.25 units of Taq DNA polymerase, 0.3 μl Nonidet P40 and about 100 ng of total DNA. The PCR conditions for both fragments are: initial denaturation at 92°C for 2 min, 30 cycles of 15 sec denaturation at 92°C, 35 sec annealing at 63°C and 10 min primer extension at 72°C followed by a final primer extension step at 72°C for 10 min. The products were sequenced by primer walking.

**Table 6:** Amplification primers used in this study

**Species**	**Primer**	**Sequence**
Both species	HCO 2198^a^	TAAACTTCAGGGTGACCAAAAAATCA
	LCO 1490^a^	GGTCAACAAATCATAAAGATATTGG
	16S Fmwg^b^	CTCGCCTTTTAWCAAAAACAT
	16S Rmwg^b^	ACGCCGGTCTKAACTCAG
	12Sai^c^	AAACTAGGATTAGATACCCTATTAT
	12Sbi^c^	AAGAGCGACGGGCGATGTGT
		
*Acanthocardia tuberculata*	Ac16Flong	GAAGCTTAAACAGTGGGACTGTTCGTCC
	Ac16Rlong	CCTATAAGACAGCTATTCCATCGTCAAC
	AcHCOrev	CGGTGAATTCATAAGATTCCAATGCTACC
	AcLCOrev	GAGCATGGTTATGTAGGCATGAAGATG
		
*Hiatella arctica*	Hi12Flong	ACCTTCAATAGCTGATCTCTACCCCAGG
	Hi12Rlong	GCATCATATCCTGTAGGGGAACTTGGCC
	Hi16Flong	AAGCTACCACGGGATAACAGCGTG
	Hi16rlong	CACGTCAACCCCTTCTTCCTAGACTTC

^a^Folmer et al. [47]

^b^modified after Palumbi et al. [48]

^c^Simon et al. [49]

### Data analysis

Protein coding genes were analysed by the Open Reading Frame Finder [32] using the invertebrate mitochondrial code. Protein and rRNA genes were identified by their similarity to published gene sequences by BLAST searches [33]. The tRNA genes are usually too little conserved for BLAST hits. Some of them were identified by tRNA-scan SE Search Server [34] and DOGMA [35], others could only be reckognised by manually folding intergenic sequences to cloverleaf structures with anticodons. Codon usage analysis was performed by CodonW version 1.3 [36]. The whole sequence was tested for potentially tandem repeats by TANDEM REPEAT FINDER, Version 4.0 [37]. The hydrophobicity profiles for the *atp8 *genes are generated using the general method of Kyte and Doolittle [38] with BIOEDIT version 7.0.5 [39].

### Phylogenetic analysis

Deduced amino acid sequences were aligned with CLUSTAL X 1.83 [40] at default settings followed by manual correction. The nucleotide alignment was based on the amino acid alignment. All protein coding and the *rrnL *gene sequences of 24 molluscs (10 bivalves, 4 cephalopods, 2 scaphopods, 7 gastropods and 1 polyplacophore [tab 6]) were concatenated in a single Nexus file. Three annelids and one brachiopod served as outgroups. Separate analyses were run with all positions and with hypervariable positions excluded with GBLOCKS 0.91 [41]. We used PAUP* 4.0b10 [42] for equally weighted parsimony analyses with the heuristic search option and 50 random addition sequences with TBR branch swapping. Bootstrap support was assessed by 10.000 (amino acid data) or 20.000 replicates (nucleotide data) with three random addition sequences each. Bayesian inference was performed with MRBAYES 3.1 [43,44] on the Schrödinger II cluster of the Univ. Vienna computing facility under the Mtrev+Γ+I substitution model for the amino acid data set. The AIC criterion implemented in MODELTEST 3.06 [45] returned the GTR+Γ+I model as most appropriate for the nucleotide data set. We used separate and unlinked partitions for each gene and 2 × 4 chains of 5 × 10^5 ^generations, sampling every 100^th ^tree. Burnin estimation by lnL and convergence diagnostics were used as implemented in the software. We excluded 3^rd ^codon positions from the nucleotide analyses to reduce phylogenetic noise due to substitution saturation. Trees were visualized with TREEVIEW 1.6.6 [46].

## Competing interests

The author(s) declare that they have no competing interests.

## Authors' contributions

HD carried out the genome sequencing and annotation, contributed to the phylogenetic analyses and drafted parts of the manuscript. GS designed the study, collected the animals, carried out the phylogenetic analyses, drafted parts of the manuscript and is responsible for the final editing.
